# The Effect of Interpersonal Psychotherapy and other Psychodynamic Therapies versus ‘Treatment as Usual’ in Patients with Major Depressive Disorder

**DOI:** 10.1371/journal.pone.0019044

**Published:** 2011-04-27

**Authors:** Janus Christian Jakobsen, Jane Lindschou Hansen, Erik Simonsen, Christian Gluud

**Affiliations:** 1 Copenhagen Trial Unit, Centre for Clinical Intervention Research, Department 3344 Rigshospitalet, Copenhagen University Hospital, Copenhagen, Denmark; 2 Psychiatric Research Unit, Copenhagen University Hospital and Region Zealand, Roskilde, Denmark; University Paris Descartes, France

## Abstract

**Background:**

Major depressive disorder afflicts an estimated 17% of individuals during their lifetimes at tremendous suffering and costs. Interpersonal psychotherapy and other psychodynamic therapies may be effective interventions for major depressive disorder, but the effects have only had limited assessment in systematic reviews.

**Methods/Principal Findings:**

Cochrane systematic review methodology with meta-analysis and trial sequential analysis of randomized trials comparing the effect of psychodynamic therapies versus ‘treatment as usual’ for major depressive disorder. To be included the participants had to be older than 17 years with a primary diagnosis of major depressive disorder. Altogether, we included six trials randomizing a total of 648 participants. Five trials assessed ‘interpersonal psychotherapy’ and only one trial assessed ‘psychodynamic psychotherapy’. All six trials had high risk of bias. Meta-analysis on all six trials showed that the psychodynamic interventions significantly reduced depressive symptoms on the 17-item Hamilton Rating Scale for Depression (mean difference −3.12 (95% confidence interval −4.39 to −1.86;P<0.00001), no heterogeneity) compared with ‘treatment as usual’. Trial sequential analysis confirmed this result.

**Discussion:**

We did not find convincing evidence supporting or refuting the effect of interpersonal psychotherapy or psychodynamic therapy compared with ‘treatment as usual’ for patients with major depressive disorder. The potential beneficial effect seems small and effects on major outcomes are unknown. Randomized trials with low risk of systematic errors and low risk of random errors are needed.

## Introduction

According to the WHO, major depressive disorder is the second largest healthcare problem worldwide in terms of illness induced disability [1]. Major depressive disorder afflicts an estimated 17% of individuals during their lifetimes at tremendous costs to the individual and society [2], [3], and roughly a third of all depressive disorders take a chronic course [4], [5]. Compared to other medical disorders, depressive illness causes the most significant deterioration in individual life quality [6]. Approximately 15% of depressive patients will commit suicide over a 10–20 year period [7].

Antidepressant medication remains the mainstay in the treatment of depression [8]. However, meta-analyses have shown that the new antidepressants only obtained beneficial effect in severely depressed patients and that this effect was clinically small [9], [10]. Antidepressants are, however, known to decrease the risk of relapse [11]. The benefits of antidepressant medication seem to be limited and this raises the question if there are other effective treatments for this serious illness?

Psychodynamic therapies origin back to Freud [12]. In some health-care systems it is currently the most commonly used form of psychotherapy [13]. Interpersonal psychotherapy is generally considered as one of the most evidence-based therapies for depression [13]. Interpersonal psychotherapy originates from classical psychodynamic therapy [14], and although interpersonal psychotherapy has integrated elements from other psychotherapies it is generally regarded as a contemporary form of psychodynamic therapy [14], [15]. We have only been able to identify one relevant meta-analysis examining the effects of psychodynamic therapies versus ‘treatment as usual’ for major depressive disorder [16]. The authors found that psychodynamic therapy is more effective than ‘treatment as usual’ for depression [16]. However, the meta-analysis did not include thorough assessment of bias risk in the included trials, did not include trials using interpersonal psychotherapy as experimental intervention, and did not employ trial sequential analysis or other methods to reduce the risk of random errors [17]–[19]. We therefore embarked on a systematic review using Cochrane methodology to assess the effect of interpersonal psychotherapy and other psychodynamic therapies versus ‘treatment as usual’ [20]. We used assessment of bias risk to reduce systematic errors [20], and trial sequential analysis to reduce the risk of random errors [17]–[19].

## Methods

We conducted our systematic review of randomized clinical trials involving meta-analysis [20] and trial sequential analysis [18], [19], [21] to answer the question: what are the beneficial and harmful effects of psychodynamic therapies versus ‘treatment as usual’ in the treatment of major depressive disorder?

For details regarding the methodology please consult our protocol published on our website (www.ctu.dk) in February 2010 before we began data extraction and analysis [22].

In short, we included all randomized clinical trials comparing the effects of interpersonal psychotherapy or other psychodynamic therapies versus ‘treatment as usual’ - irrespective of language, publication status, publication year, and publication type based on searches in The Cochrane Library's CENTRAL, MEDLINE via PubMed, EMBASE, Psychlit, Psyc Info, and Science Citation Index Expanded. The timeframe for the search was all trials published before February 2010.

To be included participants had to be older than 17 years with a primary diagnosis of major depressive disorder. Trials were only included if the diagnosis of depression was based on one of the standardized criteria, such as DSM IV [23], ICD 10 [24], DSM III [25], or DSM III-R [26].

Co-morbidity with other psychiatric diagnoses was not an exclusion criterion. The following types of trials were excluded:

- Trials focusing on depressed participants with co-morbid serious somatic illness, e.g., myocardial infarction, multiple sclerosis, cerebral stroke, cancer, etc.
- Trials focusing on ‘late life’ depression or depression in the elderly, most often participants over 65 years.
- Trials focusing on pregnancy-related depression, e.g., postpartum depression, postnatal depression, etc.
- Drug or alcohol dependence-related depression.

These exclusions were conducted because we expect participants in such trials to respond differently to standardized psychotherapy than other depressed patients, and these types of depressed patients are traditionally examined in separate trials [27]–[30].

### Interventions

To be included the trials had to use at least one of the following interventions:

- Trials using interpersonal psychotherapy [14], [15].
- Psychotherapeutic methods based on one of the classic developers of psychodynamic therapies such as Sifneos, Malan, Mann, Davanloo, or Luborsky [31].
- The notions of transference and counter-transference (raising awareness of the therapeutic relationship) [32].

Furthermore, the trials had to present a treatment manual and had to document adherence to the treatment manual for the interventions to be classified as ‘adequately defined’. All other trials that used interventions classified as ‘interpersonal’, ‘psychodynamic’, or ‘dynamic’ were included, but the interventions were classified under ‘not adequately defined’.

For ‘treatment as usual’ control interventions we accepted any non-specific treatments described as: ‘treatment as usual’, ‘standard care’, or ‘clinical management’. To be included the ‘treatment as usual’ condition had to include some kind of non-specific supportive treatment.

Trials comparing psychodynamic therapies versus ‘treatment as usual’ as add-on therapy to any co-intervention were included only if these co-interventions were described and administered similarly to the different intervention groups.

Two of the review authors (JJ and JLH) independently selected relevant trials. If a trial only was identified by one of the two, it was discussed whether the trial should be included. Excluded trials were entered on a list, stating the reason for exclusion.

### Data extraction

Data were extracted for trial design, bias risk, and outcomes independently by two authors (JJ and JLH). Disagreements were resolved by discussion or through arbitration (CG). We used the instructions in The Cochrane Handbook for Systematic Reviews of Interventions [20] in our evaluation of the methodology and hence bias risk of the included trials. We assessed the bias risk in respect to generation of the allocation sequence, allocation concealment, blinding, intention-to-treat analysis, drop-outs, reporting of outcome measures, economic bias, and academic bias. These components enable classification of the included trials into trials with ‘low risk of bias’ or with ‘high risk of bias’. The trials were overall classified as ‘high risk of bias’ if one or more of the above components was ‘uncertain’ or ‘high risk of bias’ [20], [33]–[36]. This classification is important because trials with ‘high risk of bias’ may overestimate positive intervention effects and underestimate negative effects [20], [33], [34], [36], and we wanted to relate the validity of our results to the risk of bias in the included trials.

### Primary outcome measures

#### Depressive symptoms

Our primary outcome was the mean value of the Hamilton Rating Scale for Depression (HDRS) [37], Becks Depression Inventory (BDI) [38], or Montgomery-Asberg Depression Rating Scale (MADRS) [39] at follow-up. We included data based on the total number of randomized patients (intention-to-treat analysis) if these data were reported. We planned to estimate the therapeutic follow-up responses at two time points:

- At cessation of treatment: The trials original primary choice of completion date was used. This was the most important outcome measure time point in this review.
- At maximum follow-up.

#### Adverse events

We classified adverse events as serious or non-serious. Serious adverse events were defined as medical events that are life threatening; result in death; disability or significant loss of function; that cause hospital admission or prolonged hospitalization; a hereditary anomaly; or fetal injury [40]. All other adverse events (that is, events that have not necessarily had a causal relationship with the treatment, but that resulted in a change in- or cessation of the treatment) were considered as non-serious events.

#### Quality of life

We included any measure of quality of life, noting each assessment measure.

### Secondary outcome measures

#### Participants without remission

The proportion of participants not having achieved remission. We included data based on the total number of randomized participants (intention-to-treat analysis) - if at all possible. If the results were not based on the total number of participants, we preformed an intention-to-treat analysis assuming that the participants not included in the results did not achieve remission [20]. We pragmatically defined remission as HDRS of less than 8, BDI less than 10, or MADRS less than 10 [37]–[39].

#### Participants with suicidal inclination

Number of suicide inclination, suicide attempts, or suicides.

### Statistical methods

This meta-analysis was undertaken according to the recommendations stated in The Cochrane Handbook for Systematic Reviews of Interventions [20]. In analyzing continuous outcomes with both fixed-effect and with random-effects models, we used the mean difference (MD) with a 95% confidence interval. We used RevMan version 5.0 [41]. We did not use ‘standardized mean difference’ so each outcome measure was analyzed separately. We did not adjust the outcome variables at follow-up according to the baseline values [20].

We used the odds ratio with a 95% confidence interval to estimate intervention effects on dichotomous outcomes with both fixed-effect and with random-effects models [41].

For primary outcome measures, we also conducted trial sequential analyses. In order to calculate the required information size and the cumulative Z-curve's eventual breach of relevant trial sequential monitoring boundaries [17], [18], the trial sequential analysis was based on a type I error of 5%, a beta of 10% (power of 90%), the variance of all the trials (as no trial had low risk of bias), and a minimal relevant difference of 2 points on the HDRS.

## Results

### Search results

Our primary literature search identified 3212 publications. According to our protocol [22] we excluded 3170 publications either because they did not relate to psychodynamic therapies and major depressive disorder, or because they were not randomized trials comparing psychodynamic therapies versus ‘treatment as usual’. 2831 of the 3170 were excluded on the basis of the title or abstract and 339 of the 3170 were excluded on the basis of the full publication.

Further 25 publications [42]–[66] were excluded because the trial participants or the interventions did not meet our inclusion criteria.

#### Included trials

We identified 17 publications [56]–[58], [67]–[80] on six trials [56], [67], [69], [75], [79], [80], randomizing a total of 648 participants (Figure S1). The experimental interventions were by the trialists classified as ‘interpersonal psychotherapy’ in five trials [56], [69], [75], [79], [80] and as ‘psychodynamic psychotherapy’ in one trial [67].

Only three of the trials [67], [75], [80] used an intervention that we classified as ‘adequately defined’. We classified the therapists’ level of experience and/or education one trial as ‘high’ [75], in two trials as ‘intermediate’ [67], [69], and in the last three as ‘unclear’ [56], [79], [80]. One trial used a combination of group therapy and individual therapy [69], the remaining five used only individual therapy [56], [67], [75], [79], [80].

The duration and the extend of the psychotherapy varied in the different trials from five weeks of treatment [69] to 16 weekly sessions followed by four monthly sessions [79].

The form and extend of the ‘treatment as usual’ interventions varied greatly between all of the included trials (Table 1).

**Table 1:** Characteristics of the included trials.

Trials	Participants (randomized)	Interventions	Outcomes & notes
DiMascio et al., 1979	48	Interpersonal psychotherapy (individual 16 weeks) versus supportive psychotherapy ‘on demand’ (up to one monthly session)	Raskin Depression Scale, HDRS
Elkin et al., 1989	125	Interpersonal psychotherapy (individual 16–20 weeks) versus pill-placebo and clinical management (support, encouragement and advice if necessary)	HDRS, BDI, remission HDRS (<7)
Schulberg et al., 1996	185	Interpersonal psychotherapy (16 weekly individual sessions followed by 4 monthly sessions) versus physicians usual care (various procedures commonly used by primary care physicians)	HDRS and remission HDRS (<8)
Burnand et al., 2002	90	Psychodynamic psychotherapy (individual sessions for 10 weeks) and 125 mg clomipramine versus supportive care (individual sessions for 10 weeks) and 125 mg clomipramine	HDRS, days of hospitalization, hospitalizations, lost work days, and treatment failure (major depressive disorder at 10 weeks)
Schramm et al., 2007	130	Interpersonal psychotherapy (individual and group for 5 weeks) and antidepressants (sertralin, amitriptyline) versus clinical management (3 weekly psychoeducative and supportive sessions for 5 weeks) and antidepressants (sertralin, amitrityline)	HDRS, BDI remission (HDRS <8). Participants were inpatients
Swartz et al., 2008	65	Interpersonal psychotherapy MOMS (9 individual sessions) versus treatment as usual (given referrals to mental health clinics and told to seek treatment)	HDRS, BDI. IPT MOMS differs from standardized IPT: shorter, brief behavioral strategies, specific strategies to assist mothers in managing psychiatrically ill offspring

Two trials used antidepressants in both intervention groups [67], [69]. Burnand et al. used clomipramine [67] and Schramm et al. used sertralin and amitriptyline [69]. The antidepressant medicine was delivered similarly in the experimental and control groups in both trials.

DiMascio et al. examined the effect of interpersonal psychotherapy versus ‘non-scheduled treatment’ [56]. The participants were assessed with HDRS and The Raskin Depression Scale [81]. The results at end of treatment show a significant effect of Interpersonal psychotherapy compared to ‘non- scheduled treatment’, but no significant difference was found at one-year follow-up. However, the trial did not report the SD for the mean values. We have written to the authors requesting the necessary data - but we have received no answer. Therefore, we have not been able to include the results from this trial in the following analysis.

Table 1 summarizes the characteristics of the six included trials.

#### Bias risk

We assessed all six trials [56], [67], [69], [75], [79], [80] as having ‘high risk of bias’ due to unclear or inadequate components as described in table 2.

**Table 2:** Risk of bias.

	Allocation sequence generation?	Allocation concealment?	Intention to treat analysis?	Blinding ofoutcomeassessors?	Comparability of drop-outs in intervention groups?	Free of selective outcome measure reporting?	Free of economic bias?	Free of academic bias?	Overall bias assessment
DiMascio et al., 1979	Unclear	Unclear	No	Yes	No	Unclear	Unclear	Unclear	High risk of bias
Elkin et al., 1989	Unclear	Unclear	No	Unclear	No	Yes	Yes	Unclear	High risk of bias
Schulberg et al., 1996	Unclear	Unclear	Yes	Yes	Unclear	Unclear	Unclear	Unclear	High risk of bias
Burnand et al., 2002	Unclear	Unclear	No	No	Yes	Unclear	Unclear	Unclear	High risk of bias
Schramm et al., 2007	Yes	Unclear	No	Yes	Unclear	Unclear	Yes	Unclear	High risk of bias
Swartz et al., 2008	Unclear	Unclear	No	Unclear	Unclear	Unclear	Yes	Unclear	High risk of bias

### Effects of psychodynamic therapy

#### Primary outcome measures

Five trials assessed HDRS as a continuous outcome measure at the end of treatment [67], [69], [75], [79], [80]. Three trials also assessed BDI [69], [75], [80].

#### HDRS

**Figure 1:**
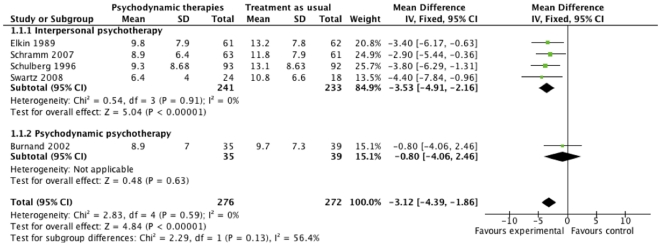
The effect of psychodynamic therapies at cessation of treatment on Hamilton Rating Scale for Depression.

Meta-analysis with fixed-effect model on the HDRS data from the five trials [67], [69], [75], [79], [80] shows that psychodynamic therapies at cessation of treatment significantly reduced depressive symptoms compared with ‘treatment as usual’. We found a mean difference on -3.12 HDRS (95% CI −4.39 to −1.86; P<0.00001, I^2^ = 0). The I^2^ statistic describes the percentage of variation across trials that are due to heterogeneity rather than chance. Sub-analysis with fixed-effect model on the HDRS-data from the four trials assessing interpersonal psychotherapy [69], [75], [79], [80] also showed a similar reduction compared with ‘treatment as usual’ (P<0.00001). However, the results from the one trial assessing psychodynamic psychotherapy [82] did not show any significant difference in effect (P = 0.63) (Figure 1).

Two of the trials reported assessment data on the HDRS after the cessation of treatment [69], [80]. Schramm et al. assessed the participants at 12 months after cessation of treatment [69]. Swartz et al. assessed at nine months after the beginning of treatment [80]. Meta-analysis with fixed-effect model on these data showed a mean difference on −4.61 HDRS (95% CI −6.98 to −2.24; P<0.0001, I^2^ = 0) in favor of psychodynamic therapies. Both trials assessed interpersonal psychotherapy.

We performed a ‘test of interaction’ [83] to analyze if the effect of two kinds of psychodynamic therapy differed between the three trials assessing ‘interpersonal psychotherapy’ [69], [75], [79], [80] and the one trial assessing ‘short psychodynamic supportive psychotherapy’ [67]. ‘Test of interaction’ showed no significant difference (P = 0.13), indicating that the effects of these two types of psychodynamic therapy do not seem to differ.

**Figure 2:**
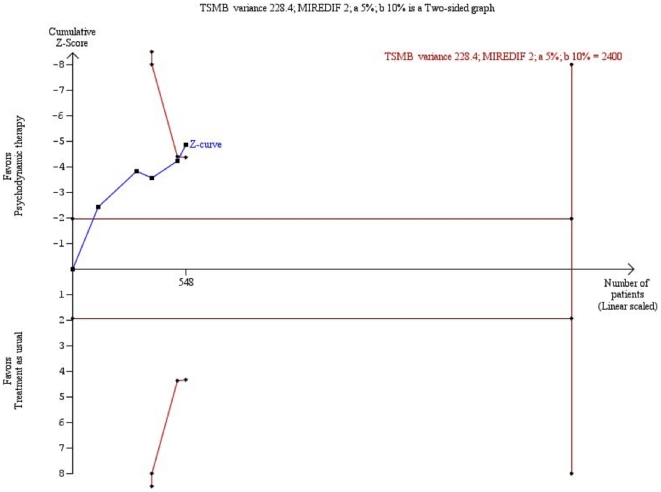
Trial sequential analysis of the cumulative meta-analysis of the effect of psychodynamic therapies versus ‘treatment as usual’ for major depressive disorder. The required information size of 2400 is calculated based on an intervention effect compared with ‘treatment as usual’, of 2 points on the HDRS, a variance of 228.4 on the mean difference, a risk of type I error of 5%, and a power of 90%. Even with these presumptions, the cumulated Z-curve (blue curve) crosses the trial sequential monitoring boundaries (red inner sloping lines) implying that there is firm evidence for a beneficial effect of psychodynamic therapies compared with ‘treatment as usual’.

Trial sequential analysis on the HDRS-data also showed a significant beneficial effect of psychodynamic therapy compared with ‘treatment as usual’ (figure 2).

#### BDI

Meta-analysis with fixed-effect model on the data from the three trials [69], [75], [80] reporting results on the BDI at cessation of treatment were in agreement with the results from the HDRS (mean difference on −3.09 BDI (95% CI −5.35 to −0.83; P = 0.007, I^2^ = 0). All three trials assessed interpersonal psychotherapy.

Meta-analysis with fixed-effect model on the data from the two trials [69], [80] reporting results on the BDI at follow-up were in agreement with the results from HDRS (mean difference on −5.54 BDI (95% CI −9.24 to −1.85; P = 0.001, I^2^ = 0).

#### Adverse events

Burnand et al. reported numbers of hospitalizations, days of hospitalization, and lost workdays in the different intervention groups [67]. They found significantly fewer of these events in the intervention group. Schramm et al. found a non-significant higher tendency for participants in the ‘treatment as usual’ group to be hospitalized after end of treatment [69]. Finally, DiMascio et al. included records on hospitalizations [56]. One participant in the experimental group and two in the control group were hospitalized.

None of the remaining trials reported on adverse events.

#### Quality of life

None of the included trials assessed the effect on quality of life of the participants.

### Secondary outcome measures

Three trials [69], [75], [79] reported the proportion of participants without remission as a dichotomous outcome measure. We had planned to define remission as a Hamilton score of less than 8, BDI less than 10, or MADRS less than 10. However, this was not possible, so we adopted the slightly different definitions of the individual trials. Two trials defined remission as HRDS less than 8 [69], [79]. One trial defined remission in two different ways: HDRS less than 7 and BDI less than 10 [75]. In the latter trial the BDI data showed no significant difference in remission between the two intervention groups [75].

**Figure 3:**
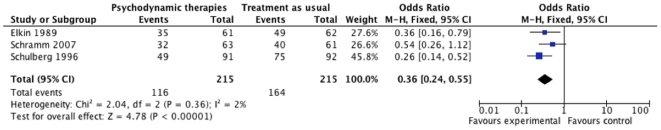
Effect of interpersonal psychotherapy on remission. Events: participants not remitting.

Meta-analysis on the HDRS-data from the three trials [69], [75], [79] showed that psychodynamic therapy compared with ‘treatment as usual’ significantly decreases the risk of no remission with an odds ratio of 0.36 (95% CI, 0.24 to 0.55; P = 0.00001, I^2^ = 2%). The number needed to treat to obtain one extra patient with remission is about four patients (95% CI, 3 to 8). All three trials assessed interpersonal psychotherapy (Figure 3).

Only two of the trials [56], [69] included records of suicide attempts and suicides. Schramm et al. reported that one participant initially treated with ‘standard care’ committed suicide 10 days after cessation of treatment [69]. No other participants attempted suicide during the trial period. DiMascio et al. reported that none of the participants had suicide attempts or committed suicide during the trial period [56].

None of the trials reported on suicide inclination.

#### Random-effects model

None of our results were changed noticeably by conducting random-effects model meta-analysis.

#### Subgroup analyses

In subgroup analyses of therapists’ level of education and experience (high versus intermediate versus unclear), type of therapy (group versus individual), and use of antidepressants as co-intervention (antidepressant co-intervention versus no antidepressant co-intervention), we found no heterogeneity in our results. This indicates that these factors do not seem to influence the effect of psychodynamic therapies.

We had also planned a subgroup-analysis according to risk of bias [22]. However, as all trials were classified as ‘high risk of bias’ it was not possible to conduct this analysis.

## Discussion

The results of our systematic review with meta-analysis and trial sequential analysis show that randomized trials with low risk of systematic errors (bias) and low risk of random errors (play of chance) are needed. Psychodynamic therapies and especially interpersonal psychotherapy might significantly reduce depressive symptoms on the HDRS and increase the probability of remission compared with ‘treatment as usual’, but due to the high risk of systematic errors (bias) we cannot make any definite conclusions. The possible benefit measured on the HDRS is presumably small. The number needed to treat to obtain one extra patient with remission may be about four patients. The impact of psychodynamic therapies on suicidality, survival, and quality of life is unknown.

It could be argued that interpersonal psychotherapy is not a psychodynamic intervention. Interpersonal psychotherapy has its theoretical roots in psychodynamic therapy but has integrated elements from other therapies [14], [15], [84]. In spite of the integrative content of interpersonal psychotherapy we chose, as it's often done in the literature, to classify interpersonal psychotherapy as a form of psychodynamic therapy [14], [85]. Furthermore, we believe that most forms of contemporary psychodynamic therapies in practice are delivered in a way similar to interpersonal psychotherapy.

### Strengths

This review has a number of strengths. Our protocol was published before we began systematic literature searches in all relevant databases, data extraction, and data analyses. Data was extracted by two independent authors minimizing the risk of inaccurate data-extraction, and we assessed the risk of bias in all trials according to the Cochrane Handbook guidelines [20]. We meta-analyzed data both with fixed-effect and random-effects models and both analyses were in agreement in all our results. Furthermore, we performed trial sequential analysis to control for random error [18], [19], [21]. The results of the trial sequential analysis confirmed the cumulative meta-analysis result.

The characteristics of the participants in the different trials, as well as the severity of the depressive symptoms differed. E.g., one trial included only inpatients [69] and another trial included depressed mothers whose children were receiving psychiatric treatment [80]. Two of the trials used antidepressants as co-intervention to psychodynamic therapies, and we included trials both assessing interpersonal psychotherapy and psychodynamic psychotherapy. Furthermore, the extent and form of the ‘treatment as usual’ condition varied greatly. We did not, however, find any heterogeneity in our analyses and found no difference on ‘test of interaction’ between interpersonal psychotherapy and psychodynamic psychotherapy. This indicates that there is a comparable treatment effect between interpersonal psychotherapy and other psychodynamic psychotherapies, between the different forms of ‘treatment as usual’, and among the different populations treated. This may make our results more generally applicable. On the other hand, few trials with few participants were included and only one trial used a psychodynamic intervention other than interpersonal psychotherapy. This decreases our power to detect any differences. Furthermore, in order to thoroughly examine a difference in effect between two interventions head-to-head comparisons are needed.

### Limitations

Our systematic review has a number of limitations. Our results are based on only six trials with a limited number of participants. Also, all six trials had high risk of bias – so our results may be questionable. Only three of the trials used an intervention that we classified as ‘adequately defined’, i.e., using and documenting the use of a therapeutic manual. In clinical trials it is imperative that the interventions are adequately defined and described [86]. Factors like personal style, communication skills, and personality of the therapist evidently will influence the way psychotherapy is delivered [87]. It is difficult to describe and control for these subjective factors, and this makes it even more important to relate the therapy to a treatment manual. Otherwise it is unclear what kind of intervention the participants were receiving, and it is difficult to apply any result in clinical practice. Moreover, a number of subgroups of depressed patients were not included in the trials of this review. These subgroups may react differently to psychotherapy and of course our review cannot be generalized to other than the included patient groups.

None of the trials reported measures of quality of life. Outcome measures of quality of life are generally not standardized and thoroughly individually validated [88]. The use of standardized outcome measures for quality of life in research has been limited by difficulties in administering and scoring quality of life [88], but quality of life can be used as a valid outcome measure [35], [88].

Typically, adverse events are not reported as thoroughly as beneficial outcome measures [89], and only two of the included trials included records of numbers of suicides and suicide attempts, and only three trials reported on some adverse events. Some psychological interventions might have harmful effects. Psychological debriefing for preventing post-traumatic stress disorder is one example [90]. Debriefing has in some clinical trials showed to have a harmful effect [90]. Possible harmful effects of this kind of therapy are therefore not thoroughly examined.

### Implications

Our results show that the possible benefit from this relatively extensive treatment compared with ‘treatment as usual’ was only a few points on the HDRS. From a clinical point of view it could be argued that this possible benefit is not clinically relevant - especially if you relate this mean difference to the extent and length of the intervention. On the other hand, our analyses demonstrate that the number needed to treat to obtain one extra patient in remission was only about four patients. The latter estimate was based on only two trials, which primarily defined remission as Hamilton score under a given value.

The HDRS might not be a useful instrument to quantify the effect of psychodynamic therapies. Other assessment methods could demonstrate a more substantial effect of any given intervention for depression. Furthermore, severity of depression as measured by the total HDRS score has failed to predict suicide attempts [91], and some publications have questioned the usefulness of the HDRS and concluded that the scale is psychometrically and conceptually flawed [92]. The two other outcome measures often used to assess depressive symptoms, MADRS and BDI, probably correspond to HDRS [93], [94]. The HDRS has during 40 years been the gold standard to quantify depressive symptoms in clinical trials [92]. There may be a need for other assessment methods.

A recently published meta-analysis examined the effect of short-term psychodynamic psychotherapy for depression [16]. As mentioned in the above, the meta-analysis did not include thorough assessment of bias risk in the included trials, did not include trials using interpersonal psychotherapy as experimental intervention, and did not employ trial sequential analysis or other methods to reduce the risk of random errors [17]–[19]. However, the results showed a significant effect of short-term psychodynamic psychotherapy on depressive symptoms and this result supports the validity of our results in the present systematic review.

Future research should focus on comparing different forms of manualized psychotherapy - or comparing psychodynamic therapy with other treatments for depression. First and foremost such trials should be conducted with low risk of bias and low risk of random errors. Such trials should also report on adverse events, suicide inclination, suicide attempts, and numbers of suicides. There may be a need for a new gold standard assessment method other than HRDS to assess depressive symptoms, and if possible more effective interventions for depression must be developed.

### Conclusions

Randomized trials with low risk of systematic errors (bias) and low risk of random errors (play of chance) are needed. Psychodynamic therapy, and especially interpersonal psychotherapy, might be an effective intervention for major depressive disorder compared with ‘treatment as usual’, but the possible treatment effect measured on the HDRS is small. The impact of psychodynamic therapies on suicidality, survival, and quality of life is unknown.

## Supporting Information

Figure S1 PRISMA flowchart.
(TIFF)
