# Correction: The Effect of Interpersonal Psychotherapy and other Psychodynamic Therapies versus ‘Treatment as Usual’ in Patients with Major Depressive Disorder

**DOI:** 10.1371/annotation/d74286e2-e872-4a7b-87d1-dfa230ff612d

**Published:** 2011-06-02

**Authors:** Janus Christian Jakobsen, Jane Lindschou Hansen, Erik Simonsen, Christian Gluud

The authors discovered an error in the computer program that performs the trial sequential analysis. The new results show that the variance on the Hamilton Depression Rating Scale (HDRS) results and the required information size both are reduced by a factor of two, similar to the bug in the program. This requires some changes to Figure 2. Please view the corrected Figure 2 here: 

**Figure:**
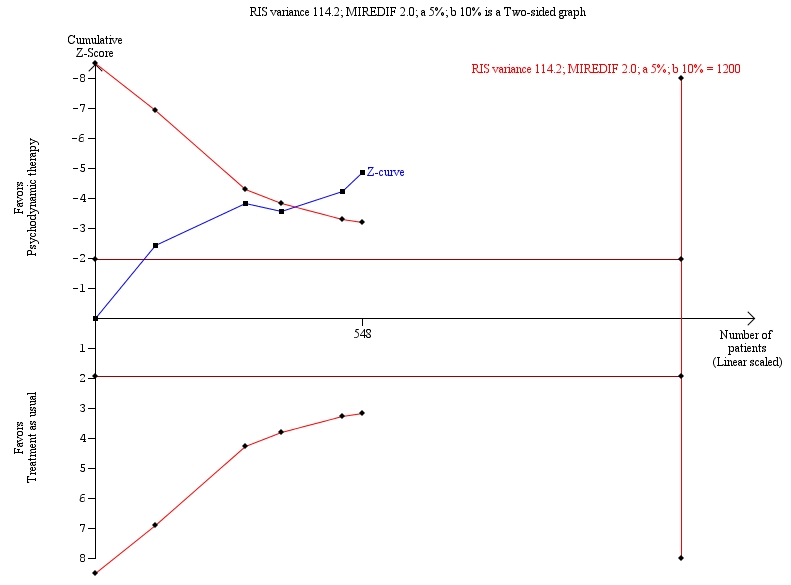


The updated Figure 2 legend and caption should read:

Trial sequential analysis of the cumulative meta-analysis of the effect of psychodynamic therapy versus 'treatment as usual'; for major depressive disorder.

The required information size (RIS) of 1200 participants is calculated based on a variance of 114.2 on the mean difference between psychodynamic therapy compared with 'treatment as usual' on the HDRS in the meta-analysis, a minimal relevant difference (MIREDIF) of 2 points on the HDRS, a risk of type I error of 5% [a], and a power of 90% (a risk of type II error of 10% [b]). Even with these presumptions, the cumulated Z-curve (the blue curve) crosses the trial sequential monitoring boundaries (the red inner sloping lines) implying that there is firm evidence for a beneficial effect of psychodynamic therapies compared with 'treatment as usual'

