# Supplementary figures and images for: The Effect of Interpersonal Psychotherapy and other Psychodynamic Therapies versus ‘Treatment as Usual’ in Patients with Major Depressive Disorder
Source: PLoS One. 2011 Apr 27;6(4):e19044. doi: 10.1371/journal.pone.0019044 (PMC3083428; doi:10.1371/journal.pone.0019044)

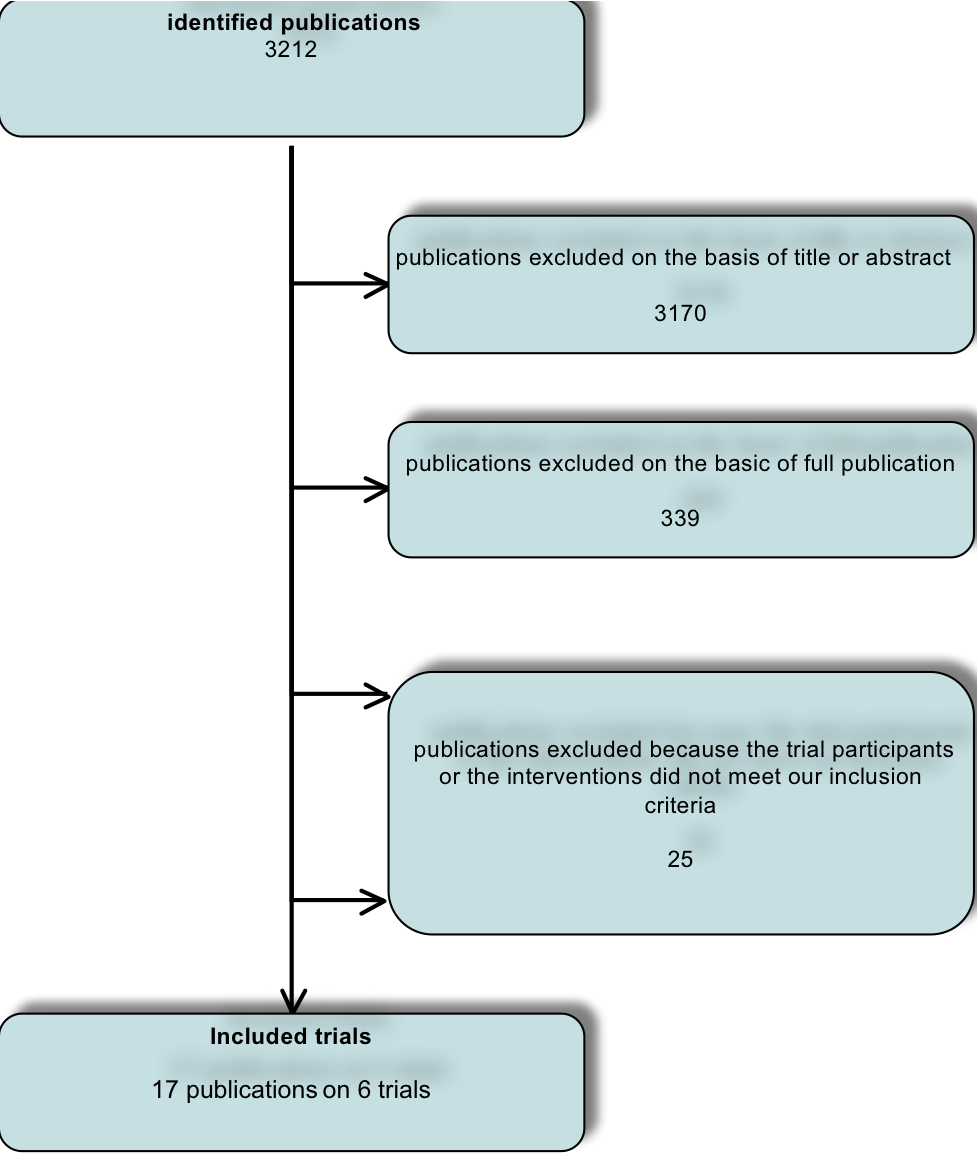

Supplement: Figure S1 — PRISMA flowchart. (TIFF) [file pone.0019044.s001.tiff]
